# Функциональное состояние эндокринной системы и минеральная плотность костной ткани в отдаленном периоде после комбинированного лечения злокачественных опухолей головного мозга в детском и молодом возрасте

**DOI:** 10.14341/probl12680

**Published:** 2021-01-08

**Authors:** О. О. Голоунина, М. Г. Павлова, Ж. Е. Белая, Е. И. Ким, И. В. Глинкина, Т. Б. Моргунова, Н. А. Мазеркина, О. Г. Желудкова, В. В. Фадеев

**Affiliations:** Первый Московский государственный медицинский университет им. И.М. Сеченова (Сеченовский Университет); Первый Московский государственный медицинский университет им. И.М. Сеченова (Сеченовский Университет); Национальный медицинский исследовательский центр эндокринологии; Национальный медицинский исследовательский центр эндокринологии; Первый Московский государственный медицинский университет им. И.М. Сеченова (Сеченовский Университет); Первый Московский государственный медицинский университет им. И.М. Сеченова (Сеченовский Университет); Национальный медицинский исследовательский центр нейрохирургии им. академика Н.Н. Бурденко; Научно-практический центр специализированной медицинской помощи детям им. В.Ф. Войно-Ясенецкого; Первый Московский государственный медицинский университет им. И.М. Сеченова (Сеченовский Университет)

**Keywords:** Злокачественные новообразования, опухоли головного мозга, медуллобластома, герминома, СТГ-дефицит, гипопитуитаризм, минеральная плотность кости, остеопороз

## Abstract

**Обоснование:**

Обоснование. Внедрение в клиническую практику стандартизированных протоколов комбинированного лечения онкологических заболеваний неизбежно приводит к развитию отдаленных последствий. Поскольку у лиц, излеченных в детском и подростковом возрасте, ожидаемая продолжительность жизни велика, своевременная диагностика и коррекция отдаленных последствий противоопухолевого лечения имеют даже большее значение, чем острые осложнения химиолучевой терапии.

**Цель:**

Цель. Изучить распространенность эндокринных нарушений, оценить распространенность и степень снижения минеральной плотности костной ткани (МПК) у лиц, перенесших комбинированное лечение злокачественных опухолей головного мозга в детском и молодом возрасте.

**Материалы и методы:**

Материалы и методы. Проведено ретроспективное исследование с участием 59 пациентов (31 мужчина; 28 женщин), перенесших в детском и молодом возрасте оперативное лечение злокачественной опухоли головного мозга с последующей лучевой терапией в объеме краниоспинального облучения в сочетании с полихимиотерапией или без нее. I группу составили 37 пациентов, которым комбинированное лечение проводилось в возрасте от 3 до 16 лет. Во II группу были включены 22 пациента, получившие лечение в возрасте от 16 до 38 лет.

**Результаты:**

Результаты. Недостаточность соматотропного гормона по результатам пробы с инсулиновой гипогликемией выявлена у 48 пациентов (81%), вторичная надпочечниковая недостаточность — у 22 (37%). Большая часть обследованных (33 пациента (56%)) не достигли целевого роста. Лечение рекомбинантным гормоном роста (рГР) получили только 5 человек из I группы. Проведенный корреляционный анализ показал, что возраст на момент лечения — основной фактор, влияющий на конечный рост (r=0,619; p<0,001). Выявлена высокая частота развития гипотиреоза (n=39 (66%)), гипогонадизма (19 женщин; 17 мужчин). По результатам DXA снижение МПК ≤-2,0 SD по Z-критерию в поясничном отделе позвоночника выявлено у 35 из 59 обследованных (59%). МПК у пациентов I группы была значимо ниже по сравнению с пациентами, получившими лечение в более старшем возрасте (p<0,001). Обнаружена умеренная корреляция между МПК в поясничном отделе позвоночника на момент обследования и уровнем эстрадиола в крови у женщин (r=0,596; p<0,05) и тестостерона у мужчин (r=0,472; p<0,05). Выявлена прямая зависимость МПК от возраста на момент заболевания (r=0,781; p<0,01).

**Заключение:**

Заключение. Полученные результаты свидетельствуют о необходимости ежегодного и пожизненного наблюдения пациентов после комбинированного лечения злокачественных опухолей головного мозга на предмет выявления отдаленных последствий лечения. Высокая распространенность остеопенических состояний определяет актуальность и необходимость проведения ранней диагностики для предотвращения дальнейшей потери костной массы, снижения прочности кости и риска переломов.

## Abstract (en)

**Background:**

Background. The implementation of standardized protocols for combined treatment of cancer into clinical practice inevitably leads to a long-term consequence.

**Aims:**

Aims. To study the prevalence of endocrine disorders, to assess the prevalence and degree of decline of bone mineral density (BMD) in individuals who have undergone combined treatment of malignant brain tumors in childhood and adolescence.

**Materials and methods:**

Materials and methods. A retrospective study was conducted with 59 young adults (31 men; 28 women) who have undergone surgical treatment of malignant brain tumour followed by radiation treatment (craniospinal radiation in combination with or without polychemotherapy). Group I consisted of 37 patients, who were treated between the ages of 3 and 16 years. Group II included 22 patients who received treatment between the ages of 16 and 38 years.

**Results:**

Results. GH deficiency according to the results of the insulin hypoglycemia test was diagnosed in 48 patients (81%), 22 ­patients had secondary adrenal insufficiency (37%). The majority of those examined (33 patients (56%)) did not achieve the target growth. Only 5 people from I group was treated with recombinant GH. Correlation analysis demonstrates that age of treatment is the main factor affecting final growth (r=0,619, p<0,001). Many cases of hypothyroidism (n=39 (66%)) and hypogonadism (19 women; 17 men) were detected. According to the DXA, a decrease of BMD ≤-2.0 SD (Z-score) in L1–L4 was found in 35 of 59 patients (59%). The BMD in the I group was significantly lower than in patients treated at an older age (p<0.001). A moderate correlation was discovered between BMD in L1–L4 at the time of examination and the level of estradiol in women (r=0.596, p<0.05) and testosterone in men (r=0.472, p<0.05). Direct correlation between BMD and age of diagnosis was revealed (r=0.781, p<0.01).

**Conclusions:**

Conclusions. The results show that patients need to be monitored annually and for life after the combined treatment of malignant brain tumors in order to detect the long-term effects of the treatment. The high incidence of osteopenic conditions determines the relevance and need for early diagnosis to prevent further bone loss, reduced bone strength and the risk of fractures.

## ОБОСНОВАНИЕ

В последние годы во всем мире стремительно растет распространенность злокачественных новообразований не только у взрослых, но и у детей. Опухоли центральной нервной системы (ЦНС) — наиболее частые солидные образования, занимающие второе место после гемобластозов в структуре онкологических заболеваний детского возраста [1]. Злокачественные опухоли головного мозга у детей в основном имеют инфратенториальную локализацию, то есть расположены в задней черепной ямке (43–63% случаев). Примерно 22,5–58% опухолей расположены субтенториально и около 7–13,5% представлены супратенториальными образованиями [2].

Опухоли ЦНС представляют собой большую гетерогенную группу новообразований, разнообразие гистологических вариантов которых зависит от возраста пациента. Медуллобластома (МБ) — наиболее распространенная злокачественная опухоль головного мозга у детей (15–20% всех первичных опухолей ЦНС). Особенностью МБ является высокая склонность к метастазированию по ликворным путям. Системные метастазы выявляются в 5% случаев, часто поражая кости [3]. Несколько реже у детей встречаются злокачественные глиомы (мультиформная глиобластома, анапластическая астроцитома), эпендимомы и герминомы. В структуре опухолей низкой степени злокачественности преобладает пилоидная астроцитома [4]. Лишь небольшую долю внутричерепных образований составляют опухоли шишковидной железы, представляющие собой объемные образования срединных структур ЦНС и часто сопровождающиеся гидроцефалией. Среди опухолей шишковидной железы наиболее распространены пинеобластомы и герминомы [5]. Последние могут также развиваться в переднем отделе гипоталамуса или на дне III желудочка. Такие опухоли часто сопровождаются клинической триадой: несахарным диабетом, гипофизарной недостаточностью и нарушениями зрения. Внутричерепные герминомы имеют склонность к локальному распространению, инфильтрируют гипоталамус и метастазируют в спинной мозг через цереброспинальную жидкость. Экстракраниальные метастазы образуются редко [2].

За последние десятилетия достигнуты значительные успехи в лечении онкологических заболеваний у детей. Внедрение в клиническую практику стандартизированных протоколов комбинированного лечения (операция, лучевая терапия (краниальное (КО) или краниоспинальное облучение (КСО)) и полихимиотерапия (ПХТ)) позволило увеличить общую и безрецидивную выживаемость до 70–80% [6][7]. Однако подобная терапия неизбежно приводит к широкому спектру нежелательных эффектов и отдаленных последствий, в том числе оказывает негативное влияние на эндокринную систему и костную ткань [8]. Поскольку у лиц, излеченных в детском и подростковом возрасте от онкологического заболевания, ожидаемая продолжительность жизни велика, выявление и коррекция отдаленных последствий комбинированного лечения имеют даже большее значение, чем острые осложнения лучевой терапии и ПХТ, возникающие в первые дни или недели после воздействия.

## ЦЕЛЬ

Изучить частоту развития эндокринных нарушений, распространенность и степень снижения минеральной плотности костной ткани (МПК) у лиц, перенесших комбинированное лечение злокачественных опухолей головного мозга в детском и молодом возрасте.

## МАТЕРИАЛЫ И МЕТОДЫ

## Дизайн исследования

Проведено одноцентровое одномоментное исследование с ретроспективным анализом данных.

В исследование включены 59 пациентов (31 мужчина; 28 женщин, соотношение м:ж = 1,1:1), перенесших в детском и молодом возрасте лечение злокачественной опухоли головного мозга: у 45 пациентов была МБ, у 10 — герминативно-клеточная опухоль, у 2 — анапластическая эпендимома, у 2 — анапластическая астроцитома (рис. 1).

**Figure:**
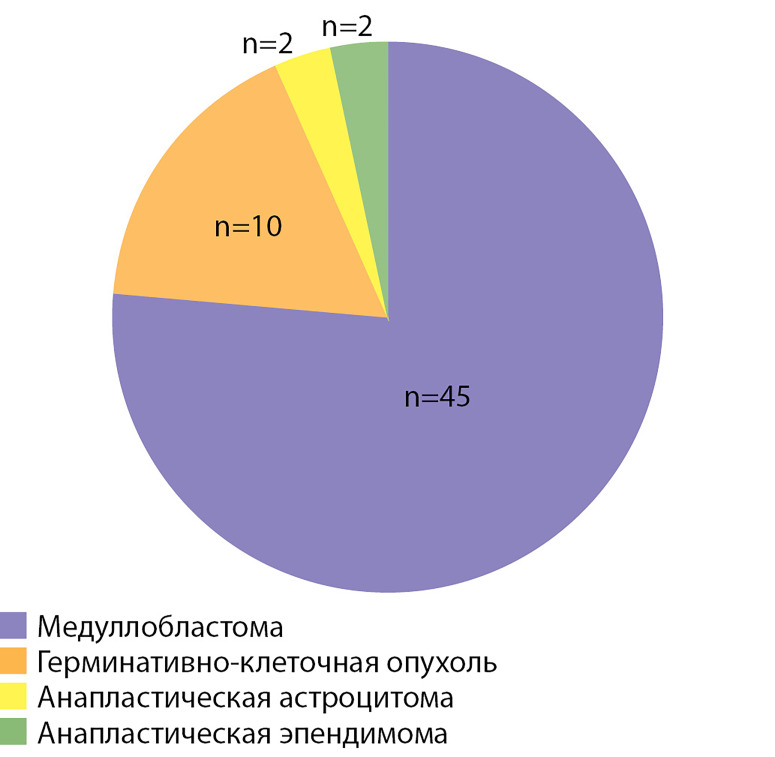
Рисунок 1. Распределение пациентов в зависимости от гистологического типа опухоли головного мозга.

## Критерии соответствия

Критерии включения в исследование: пациенты ≥18 лет, перенесшие комбинированное лечение опухоли головного мозга в детском и молодом возрасте; длительность ремиссии более 2 лет; отсутствие рецидива онкологического заболевания на момент исследования.

Критерии исключения из исследования: отсутствие стойкой ремиссии или рецидив основного заболевания; беременность; лактация; наличие тяжелой сопутствующей патологии.

## Условия проведения

Исследование проводилось на базе кафедры эндокринологии №1 Института клинической медицины им. Н.В. Склифосовского ФГАОУ ВО «Первый МГМУ им. И.М.  Сеченова» Минздрава России (Сеченовский Университет).

## Продолжительность исследования

Набор материала осуществлялся в течение 3 лет, с сентября 2017 по сентябрь 2020 гг.

## Описание медицинского вмешательства

В исследование были включены пациенты, перенесшие в детском и молодом возрасте комплексное лечение злокачественной опухоли головного мозга (оперативное лечение с последующей лучевой терапией в объеме КСО СОД (суммарная очаговая доза) 24–56 Гр в сочетании с ПХТ или без нее). ПХТ получали 56 из 59 обследованных. В 17 случаях химиотерапия проводилась по протоколу HIT-2000, 9 пациентам — по протоколу SIOP-CNS GCT-96, 1 пациенту — по протоколу PO-CNS-02. Большая часть пациентов получали ПХТ по протоколу М-2000: 9 пациентов получили 4 цикла цикловой ПХТ и 20 пациентов — 8 циклов поддерживающей ПХТ.

Клинические методы обследования включали анализ анамнестических и антропометрических данных. Всем пациентам проводились гормональные исследования, включавшие определение уровней тиреотропного гормона (ТТГ), свободной фракции тироксина (Т4 свободный), кортизола, пролактина, лютеинизирующего гормона (ЛГ), фолликулостимулирующего гормона (ФСГ), половых стероидов, ингибина В, антимюллерова гормона (АМГ), инсулиноподобного фактора роста-1 (ИФР-1), паратиреоидного гормона (ПТГ). Для диагностики соматотропной недостаточности и вторичной надпочечниковой недостаточности проводилась проба с инсулиновой гипогликемией (ИГГ). Перед проведением диагностических тестов у всех пациентов, имеющих гипотиреоз, гипогонадизм, гипокортицизм, была достигнута медикаментозная компенсация.

Женщинам проводилось ультразвуковое исследование (УЗИ) органов малого таза, мужчинам — УЗИ органов мошонки. Всем пациентам выполнялось измерение МПК методом двухэнергетической рентгеновской абсорбциометрии (dual-energy-X-ray absorptiometry, DXA).

## Основной исход исследования

Основными конечными точками исследования были функциональное состояние эндокринной системы и показатели МПК в отдаленном (от 2,5 до 12 лет) периоде после проведенного комбинированного лечения злокачественных опухолей головного мозга.

## Анализ в подгруппах

Все пациенты, включенные в исследование, были разделены на 2 группы.

В I группу вошли 37 человек (20 мужчин и 17 женщин, м:ж =1,18:1), которым лечение злокачественной опухоли головного мозга проводилось в возрасте от 3 до 16 лет. Медиана (Ме) возраста на момент обследования составила 20 лет [ 18;23 ]; Ме возраста на момент лечения — 12 лет [ 9; 14 ].

Во II группу включены 22 пациента (11 мужчин и 11 женщин, м:ж =1:1), получивших комбинированное лечение опухоли головного мозга в возрасте от 16 до 38 лет. Ме возраста на момент обследования составила 24 года [ 21; 28 ]; Ме возраста на момент лечения  — 20 лет [ 17; 25 ].

## Методы регистрации исходов

Диагноз соматотропной недостаточности подтверждался при пике выброса соматотропного гормона (СТГ) в пробе с ИГГ менее 3 нг/мл [9]. Диагноз вторичной надпочечниковой недостаточности устанавливался при уровне кортизола крови в пробе с ИГГ менее 500 нг/мл.

Диагноз первичного манифестного гипотиреоза устанавливался, если уровень ТТГ был выше 10 мМЕд/л при нормальном или сниженном уровне свободного Т4. Изолированное повышение ТТГ до 10 мМЕд/л при нормальных значениях свободного Т4 расценивалось как субклинический первичный гипотиреоз. Диагноз вторичного гипотиреоза устанавливался на основании снижения уровня свободного Т4 при нормальных или сниженных значениях ТТГ, а также нарушения суточного ритма секреции ТТГ. Гипотиреоз смешанной этиологии (далее — смешанный гипотиреоз) устанавливался при значениях свободного Т4 в пределах нижней трети референсного интервала в сочетании с незначительным повышением (до 10 МЕд/мл) уровня ТТГ и отсутствии ритма секреции ТТГ.

Показатели уровня АМГ и объема яичников использовались для оценки состояния овариального резерва у женщин. Репродуктивные нарушения у мужчин оценивались по изменению уровня гонадотропных гормонов, снижению уровня тестостерона, уменьшению объема яичек, задержке полового развития, бесплодию, эректильной дисфункции. С целью косвенной оценки сперматогенной функции использовался уровень ингибина В: снижение ингибина В менее 80 нг/мл считалось маркером нарушенного сперматогенеза.

Для оценки соответствия индивидуального роста ребенка референсным для конкретного возраста и пола данным использовался интегральный показатель SDS (standard deviation score).

Исследование крови было проведено на автоматических анализаторах Immulite 2000i (Siemens Healthcare Diagnostics Inc, Германия-США), ADVIA CentaurXP (Siemens Healthcare Diagnostics, Германия), используя твердофазный иммунометрический метод с хемилюминесцентной детекцией. Уровни ингибина В и АМГ определялись методом иммуноферментного анализа.

МПК измерялась методом DXA на аппарате Lunar iDXA (General Electric Healhcare, США) в поясничном отделе позвоночника (LI–LIV), шейке бедренной кости (Neck) и в целом в бедре (Total hip). Для оценки степени снижения МПК использовался Z-критерий. За снижение костной массы относительно возрастной нормы принимали Z-критерий ≤-2,0 SD.

## Этическая экспертиза

Исследование было одобрено локальным этическим комитетом ФГАОУ ВО «Первый МГМУ им. И.М. Сеченова» (Сеченовский Университет) Минздрава России (протокол № 25-20 от 09.09.2020 г.).

## Статистический анализ

Объем выборки предварительно не рассчитывался. Статистический анализ данных осуществлялся при помощи пакета статистических программ IBM SPSS Statistics 23 (SPSS. Inc, Chicago, IL, USA). Для представления количественных данных использована медиана (Me) с указанием межквартильного диапазона, максимальных и минимальных значений. Степень достоверности различий в независимых выборках определялась критерием Манна–Уитни. Для определения статистической значимости относительных показателей использовался критерий согласия Пирсона (χ2) (для таблиц 2х2 — в точном решении Фишера). Связь между истинно числовыми показателями анализировали при помощи расчета коэффициента корреляции Пирсона (r). Статистически достоверными считались различия при p<0,05.

## РЕЗУЛЬТАТЫ

## Объекты (участники) исследования

В настоящее исследование включены 59 пациентов после лечения злокачественной опухоли головного мозга: 31 мужчина (52,5%) и 28 женщин (47,5%). Общая характеристика пациентов представлена в таблице 1.

**Table:** Таблица 1. Клиническая характеристика пациентов, включенных в исследование

		I группа	II группа	Достоверность различий, p
Пол	мужчины	20	11	
женщины	17	11	
Возраст, лет	на момент заболевания	Ме 12 [ 9; 14]	Ме 20 [ 17; 25]	<0,001
на момент обследования	Ме 20 [ 18; 23]	Ме 24 [ 21; 28]	0,205
Ремиссия, лет	длительность на момент обследования	Ме 7,5 [ 5; 12]	Ме 3 [ 2,5; 7]	
ПХТ	проводилась	35	21	
не проводилась	2	1	
ЛТ	КО СОД	22–40 Гр	
КСО СОД	24–54 Гр	35–56 Гр	
буст на ЗЧЯ	54–56 Гр	

Примечание: ПХТ — полихимиотерапия; ЛТ — лучевая терапия; КО — краниальное облучение; КСО — краниоспинальное облучение; ЗЧЯ — задняя черепная ямка; СОД — суммарная очаговая доза.

## Основные результаты исследования

В отдаленном периоде после комбинированного лечения злокачественных опухолей головного мозга СТГ-недостаточность по результатам пробы с ИГГ выявлена у 48 (81%) из 59 обследованных, вторичная надпочечниковая недостаточность — у 22 (37%) пациентов (рис. 2). В группе пациентов, пролеченных в более раннем возрасте, несколько чаще выявлялась недостаточность гормона роста (ГР) по сравнению с теми, кто лечился в старшем возрасте (табл. 2, рис. 2). Целевого роста не достигли 33 (56%) пациента, из них 31 пациент — в I группе и только 2 — во II группе. Лечение рекомбинантным ГР (рГР) получили 5 пациентов из I группы. SDS конечного роста у них был несколько выше, чем SDS роста до лечения рГР, однако никто из пациентов не достиг целевого роста. Проведенный корреляционный анализ показал, что возраст на момент лечения — основной фактор, влияющий на конечный рост (r=0,619; p<0,001). Пациенты, перенесшие комбинированное лечение опухоли головного мозга в более старшем возрасте, чаще достигали своего целевого роста в сравнении с теми, кто лечился в младшем возрасте. Зависимости конечного роста от уровня тиреоидных гормонов, ТТГ, ЛГ, ФСГ не обнаружено.

**Figure:**
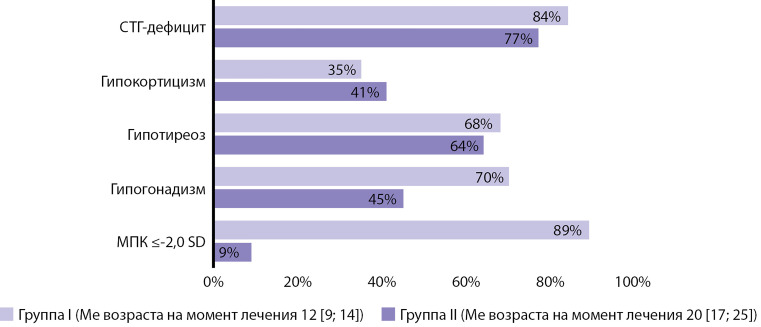
Рисунок 2. Распространенность отдаленных последствий комбинированного лечения злокачественных опухолей головного мозга.

 

**Table:** Таблица 2. Сравнение ростовых показателей, распространенности эндокринных и костных нарушений в исследуемых группах

	I группа	II группа	Достоверность различий, p
SDS конечного роста	Ме -1,6 [ -2,5; -0,2 ]	Ме 0,4 [ -0,1; 0,8 ]	<0,001
Частота СТГ-дефицита, n (%)	31 (84%)	17 (77%)	0,074
Частота гипокортицизма, n (%)	13 (35%)	9 (41%)	0,463
Частота гипотиреоза, n (%)	25 (68%)	14 (64%)	0,644
Частота гипогонадизма, n (%)	26 (70%)	10 (45%)	0,022
Частота развития остеопенических состояний, n (%)	33 (89%)	2 (9%)	<0,001

Более половины обследованных пациентов имели гипотиреоз (n=39 (66%)): у 24 верифицирован первичный (манифестный или субклинический гипотиреоз), у 8 — вторичный гипотиреоз, 7 пациентов имели смешанный гипотиреоз. Нарушение функции щитовидной железы выявлялось с одинаковой частотой в обеих группах (см. табл. 2, рис. 2).

Нарушение менструального цикла отмечалось у 19 (68%) молодых женщин после комбинированного лечения опухоли головного мозга. Аменорея выявлена у 12 женщин, из них первичная и вторичная аменорея были диагностированы у 3 и 9 женщин соответственно, однако заместительная эстроген-гестагенная терапия была назначена только 8 пациенткам. Жалобы на нарушение менструального цикла по типу олигоменореи предъявляли 7 женщин. На момент проведения исследования только у 6 (21%) женщин был регулярный менструальный цикл. Следует отметить, что в группе пациентов, которым комбинированное лечение было проведено в пубертатном возрасте, почти в два раза чаще встречались репродуктивные нарушения (см. табл. 2, рис. 2).

Уровень пролактина в пределах референсного диапазона имели 26 из 28 обследованных женщин, у 2 пациенток отмечалось повышение уровня макропролактина до 778 и 928 соответственно (109–557 мЕд/л). Уровень АМГ в пределах референсных значений наблюдался только у 9 из 28 обследованных женщин. Ме уровня АМГ составила 0,19 [ 0,4; 1,9 ]. В ходе данного исследования корреляции уровня АМГ с уровнем гонадотропных гормонов (ЛГ и ФСГ) не обнаружено, однако выявлена значимая связь между уровнем АМГ и возрастом пациентки на момент проведения ПХТ (р=0,003).

У мужчин доказанные репродуктивные нарушения имели 17 обследованных (55%). Частота развития гипогонадизма коррелировала с возрастом пациента на момент лечения и суммарным объемом яичек (r=0,529; р=0,02). Ме уровня ингибина В в обследованной когорте (n=31) составила 42 нг/мл (от 10 до 183). Наблюдалась положительная корреляция между уровнем ингибина В и объемом яичек (r=0,464; p=0,02).

Ни у одного из обследованных в анамнезе не было зарегистрировано низкотравматичных переломов. При этом снижение костной массы относительно возрастной нормы по результатам DXA (Z-критерий ≤-2,0 SD) в поясничном отделе позвоночника (LI–LIV) выявлено у 35 из 59 пациентов (59%). Установлено, что лица, пролеченные в детском возрасте, достоверно чаще имели выраженное снижение МПК в сравнении с теми, кто лечился в более старшем возрасте (p<0,001) (см. табл. 2, рис. 2). Показатели МПК в обеих группах в целом варьировали от +0,8 SD до -4,2 SD (табл. 3). Обнаружена умеренная корреляция между МПК в поясничном отделе позвоночника на момент обследования и уровнем эстрадиола в крови у женщин (r=0,596; p<0,05) и тестостерона у мужчин (r=0,472; p<0,05). Выявлена прямая зависимость МПК от возраста на момент заболевания (r=0,781; p<0,01) (рис. 3). Проекционная МПК в исследуемых участках скелета была доступна только для 14 пациентов и составила от 0,656 до 1,114 г/см2. Изменения уровня ПТГ не было ни у одного из обследованных пациентов, однако нами обнаружена отрицательная корреляция между уровнем ПТГ и МПК (r=-0,374; p=0,045).

**Figure:**
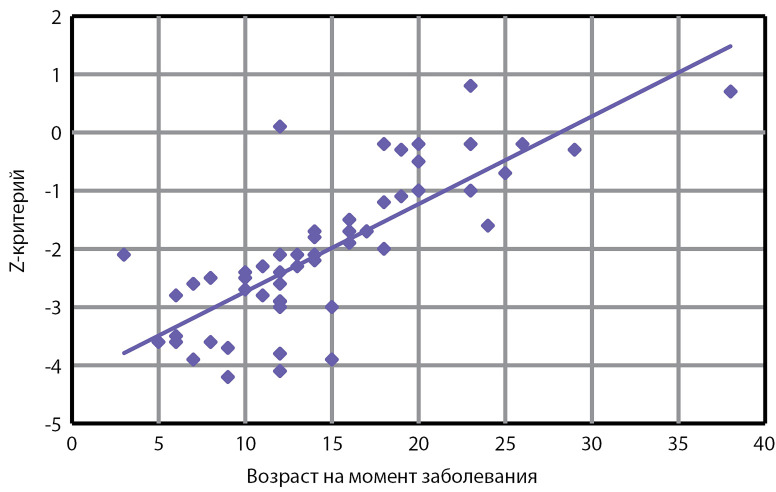
Рисунок 3. Зависимость минеральной плотности костной ткани в поясничном отделе позвоночника (LI–LIV) от возраста на момент заболевания (r=0,781; p<0,01).

 

**Table:** Таблица 3. Минеральная плотность костной ткани у пациентов после комбинированного лечения злокачественных опухолей головного мозга в детском и подростковом возрасте

	Группа I	Группа II	Достоверность различий, p
Количество пациентов	37	22	
Значения МПК LI–LIV (SD)	от -4,2 до +0,1	от -1,9 до +0,8	
Медиана	-2,75	-1,0	<0,001

## ОБСУЖДЕНИЕ

Комбинированная терапия злокачественных опухолей головного мозга является крайне агрессивным фактором и неизбежно приводит к развитию отдаленных последствий, в том числе к значительным патологическим изменениям со стороны эндокринной системы и костной ткани. В обследованной нами когорте пациентов наиболее частыми эндокринными расстройствами оказались соматотропная недостаточность, гипогонадизм и гипотиреоз различной этиологии. Нарушения со стороны эндокринной системы могут развиваться как во время лечения, так и непосредственно после его окончания, но гораздо чаще — в отдаленном периоде после завершения комбинированной терапии [10]. В первую очередь это связано с особенностями лучевой терапии. Выраженность гормонального дефицита и степень тяжести в основном зависят от суммарной дозы и области облучения, времени, прошедшего после окончания лучевой терапии, а также возраста пациента на момент проведения лечения [11].

СТГ-недостаточность является одним из первых проявлений постлучевого гипопитуитаризма [12]. Согласно литературным данным, лучевая терапия в СОД 30–50 Гр при облучении всего объема головного мозга в 50–100% случаев сопровождается развитием недостаточности ГР [13][14]. В нашем исследовании дефицит СТГ, уровень которого крайне важен, в том числе для достижения пиковой костной массы [15], также встречался очень часто — в 81% случаев (по результатам пробы с ИГГ).

Недостаточность ГР — один из важнейших факторов снижения линейного роста у детей после КО. Согласно проведенным исследованиям, заместительная терапия препаратами рГР у таких пациентов может предотвратить дальнейшее возможное снижение темпов роста [16]. Помимо этого, на динамику роста оказывает влияние возраст на момент лечения [17]. Как показало наше исследование, конечный рост обследованных, пролеченных в более позднем возрасте, оказался статистически значимо выше, как и достижение ими целевого роста. Нормальный конечный рост имеет большое влияние на качество жизни и социальную адаптацию пациентов во взрослой жизни. В связи с этим детям с диагностированным дефицитом гормона роста крайне важно своевременное (до закрытия зон роста) назначение заместительной терапии препаратами рГР. Однако длительность ремиссии для принятия решения о назначении рГР является предметом дискуссии. Общепринятым считается назначение рГР через 2 года стойкой ремиссии, особенно если лечение было проведено в детском возрасте [10]. До назначения препаратов рГР пациенты с гипопитуитаризмом должны быть компенсированы по гипокортицизму и гипотиреозу.

Одним из осложнений комбинированного лечения опухолей головного мозга является снижение МПК, представляющее сложную многофакторную проблему. Хирургическое лечение само по себе не оказывает влияния на структуру кости. Потеря костной массы обусловлена последующей лучевой терапией и ПХТ. КСО в сочетании с ПХТ приводят к дефициту СТГ и гипогонадизму, что сопровождается развитием остеопенических состояний [18]. В современных протоколах активно используются химиотерапевтические препараты, оказывающие токсический эффект, в том числе на костную ткань, за счет подавления ростовых процессов, нарушения микроархитектоники кости и снижения ее минеральной плотности [19]. Кроме того, ПХТ напрямую снижает продукцию ИФР-1 в печени, нарушая при этом его влияние на ростовую пластинку [20]. Гипогонадизм в исходе противоопухолевой терапии может привести к тяжелым формам остеопороза. Дефицит половых гормонов сопровождается повышением активности остеокластов, запуская фазу быстрой потери костной массы [21]. Недостаточное питание и мальабсорбция во время лечения влекут за собой дефицит кальция и витамина D вследствие снижения их потребления и всасывания в кишечнике. Баланс кальция в крови восстанавливается путем поступления кальция из костей, усиливая таким образом снижение МПК [22].

Развитие остеопенических состояний в отдаленном периоде лечения злокачественных опухолей головного мозга у детей было описано в сравнительно небольшом количестве зарубежных работ [23–28], однако похожих исследований в отечественной литературе найти не удалось.

В нашем исследовании снижение МПК в поясничном отделе позвоночника в отдаленном периоде наблюдения выявлено в 59% случаев. Высокая распространенность остеопенических состояний определяет актуальность и необходимость изучения данной проблемы с целью оптимизации ранней диагностики для предотвращения тяжелых осложнений в будущем. Учитывая, что у пациентов с опухолями головного мозга, особенно с локализацией в области задней черепной ямки и мозжечка, нередко наблюдается атаксия, проявляющаяся расстройствами поддержания равновесия и ходьбы, пошатыванием и нарушениями координации движений [29], в сочетании со сниженной МПК существенно увеличивается риск переломов даже в молодом возрасте.

Результаты проведенного исследования показали прямую зависимость МПК обследованных от уровня половых гормонов (эстрадиола у женщин, тестостерона у мужчин) и возраста на момент проведения лечения. Пубертатный возраст является критическим периодом в формировании костной системы, поскольку около половины пиковой костной массы формируется именно в подростковом возрасте [30]. Очевидно, что при гипогонадизме в детском и молодом возрасте нарушается набор пиковой костной массы. Недостаточное накопление костной массы в первые 20 лет жизни может привести к раннему развитию остеопороза и, как следствие, к низкотравматическим переломам у взрослых. Доказано, что в случае снижения костной массы относительно возрастной нормы на 5–10% в подростковом возрасте риск перелома шейки бедра в пожилом возрасте возрастает почти на 50% [31]. Таким образом, подавляющее большинство обследованных нами пациентов входят в группу риска по развитию тяжелых осложнений остеопороза в будущем.

Необходимо учитывать, что в норме костная масса достигает своего пика примерно в 25–30 лет, тогда как лечение опухолей головного мозга в основном приходится на детский и подростковый возраст (рис. 4). Согласно последним рекомендациям Международного общества клинической денситометрии (International Society for Clinical Densitometry, ISCD), только в случае снижения МПК ≤-2,0 SD по Z-критерию и наличия клинически значимых переломов крупных костей скелета или при наличии как минимум одного зарегистрированного компрессионного перелома тела позвонка в анамнезе, независимо от показателей DXA, можно говорить об остеопорозе и рассмотрении вопроса о назначении соответствующего лечения [32]. Подобные изменения у пациентов, перенесших противоопухолевое лечение, необходимо трактовать как вторичный остеопороз (СТГ-дефицит, гипогонадизм). При отсутствии низкотравматичных переломов диагноз «остеопороз», нередко выставляемый молодым пациентам только на основании результатов DXA, неправомочен. Можно говорить только о снижении МПК ниже ожидаемых значений.

**Figure:**
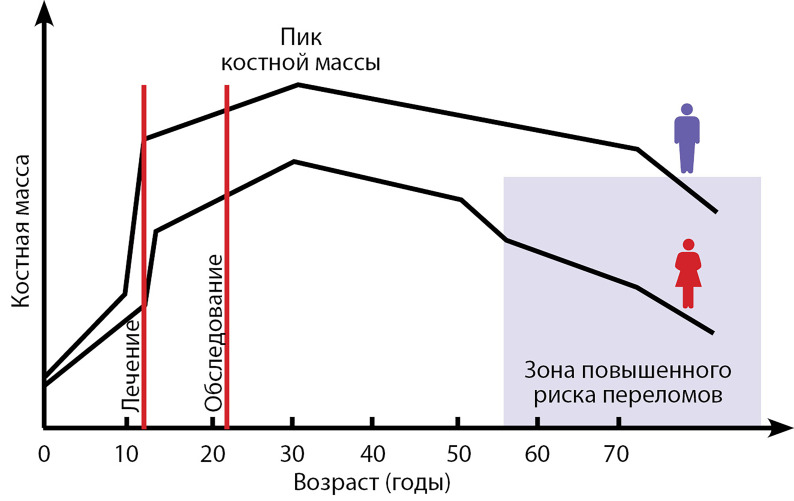
Рисунок 4. Изменение минеральной плотности кости в разные возрастные периоды [37].

Полученные результаты демонстрируют необходимость оптимизации алгоритма диагностики и своевременной профилактики патологических изменений костной ткани у пациентов после лечения злокачественных опухолей головного мозга в детском и молодом возрасте. На сегодняшний день отсутствуют официальные рекомендации по скринингу и лечению остеопороза у детей, в том числе после комбинированного лечения злокачественных опухолей головного мозга. Перспективными представляются проведение подробного анализа состояния метаболизма костной ткани, оценка качества и микроархитектоники кости, в том числе с использованием трабекулярного костного индекса (Trabecular Bone Score, TBS), позволяющего оценить микроструктуру костной ткани на основании данных, полученных в ходе проведения стандартной DXA поясничного отдела позвоночника [33].

С целью проведения ранней диагностики представляется оправданным введение обязательного измерения костной плотности. Исследование МПК методом DXA целесообразно не ранее чем через 2 года после завершения лечения основного заболевания [8]. При наличии болевого синдрома в спине необходимо направление пациента на рентгенографию грудного и поясничного отделов позвоночника (ThIV–LV) в боковой проекции.

Несмотря на высокую распространенность остеопенических состояний у пациентов, перенесших комбинированное лечение опухоли головного мозга в детском и молодом возрасте, вопрос о выборе медикаментозной терапии остается одной из наиболее актуальных и дискутабельных проблем в педиатрии. Назначение доступной на сегодняшний день в Российской Федерации антирезорбтивной терапии бисфосфонатами, деносумабом (рекомбинантное моноклональное IgG2 антитело к RANKL) [34][35], анаболической терапии терипаратидом (рекомбинантный человеческий ПТГ [rhPTH (1–34)]) нецелесообразно ввиду низкой доказательной базы по критериям безопасности и эффективности у молодых пациентов, а также наличия противопоказаний: возраст <18 лет — для назначения бисфосфонатов [34], незакрытые зоны роста и облучение в анамнезе — для терапии терипаратидом [36]. Таким образом, грамотный выбор медикаментозной терапии, продолжительности лечения является непростой и ответственной задачей, которая должна решаться индивидуально в конкретной клинической ситуации с учетом выраженности потери МПК, наличия осложнений и совокупности факторов риска. По всей видимости, своевременное назначение заместительной терапии половыми гормонами и ГР в метаболических дозах у взрослых может стать оптимальным методом повышения МПК.

## ВЫВОДЫ

1. Распространенность эндокринных нарушений после перенесенного комбинированного лечения злокачественных опухолей головного мозга в детском и молодом возрасте крайне высока, что требует обязательного участия эндокринолога в пожизненном наблюдении за данными пациентами.

2. Учитывая высокую распространенность остеопенических состояний после перенесенного комбинированного лечения злокачественных опухолей головного мозга, целесообразно проведение регулярного исследования костной плотности методом DXA после завершения лечения основного заболевания.

3. Своевременное назначение заместительной терапии половыми гормонами и ГР может стать оптимальным методом профилактики изменений костной ткани после лечения злокачественных заболеваний у молодых пациентов.

## ОГРАНИЧЕНИЯ ИССЛЕДОВАНИЯ

Определенные ограничения исследования связаны с ретроспективной оценкой имеющейся медицинской документации, одномоментным срезом данных. Принимая во внимание, что в исследование были включены пациенты с длительностью ремиссии от 2 лет, недостаточность ГР на момент проведения обследования у некоторых лиц могла еще не развиться.

## ЗАКЛЮЧЕНИЕ

В последние годы во всем мире увеличивается популяция взрослых, пролеченных в детском и молодом возрасте от онкологического заболевания, что неизбежно приводит к необходимости своевременной диагностики, адекватного лечения и, главное, профилактики отдаленных последствий химиолучевой терапии. Полученные нами данные свидетельствуют о необходимости ежегодного и пожизненного наблюдения пациентов на предмет выявления отдаленных последствий лечения. Изучение особенностей костного метаболизма после комбинированного лечения злокачественных опухолей головного мозга позволит оптимизировать раннюю диагностику для предотвращения дальнейшей потери костной массы, снижения прочности кости и риска переломов. Особенно актуальной является разработка стандартов обследования данных пациентов и схем коррекции выявленных костных нарушений. Целесообразны назначение препаратов кальция и витамина D в период ЛТ, ПХТ и сразу после их окончания, динамический контроль уровня половых гормонов, состояния фосфорно-кальциевого обмена, своевременное назначение заместительной терапии с целью компенсации выявленных нарушений, минимизации негативного влияния данных факторов на состояние МПК.
